# The transcriptional landscape of basidiosporogenesis in mature *Pisolithus microcarpus* basidiocarp

**DOI:** 10.1186/s12864-017-3545-5

**Published:** 2017-02-14

**Authors:** Maíra de Freitas Pereira, André Narvaes da Rocha Campos, Thalita Cardoso Anastacio, Emmanuelle Morin, Sérgio Hermínio Brommonschenkel, Francis Martin, Annegret Kohler, Maurício Dutra Costa

**Affiliations:** 1 Department of Microbiology/BIOAGRO, Universidade Federal de Viçosa, Viçosa, MG Brazil; 2 INRA, UMR 1136 INRA-University of Lorraine, Interactions Arbres/Microorganismes, Laboratory of Excellence ARBRE, INRA-Nancy, 54280 Champenoux, France; 3 IF Sudeste MG, Rio Pomba, MG Brazil; 4 Department of Phytopathology/BIOAGRO, Federal University of Viçosa, Viçosa, MG Brazil

**Keywords:** Gene expression, Peridiole development, Spores, Cell cycle, Fatty acid metabolism

## Abstract

**Background:**

*Pisolithus microcarpus* (Cooke & Massee) G. Cunn is a gasteromycete that produces closed basidiocarps in symbiosis with eucalypts and acacias. The fungus produces a complex basidiocarp composed of peridioles at different developmental stages and an upper layer of basidiospores free of the hyphae and ready for wind dispersal upon the rupture of the basidiocarp pellis. During basidiosporogenesis, a process that takes place inside the basidiocarp peridioles, a conspicuous reserve of fatty acids is present throughout development. While several previous studies have described basidiosporogenesis inside peridioles, very little is known about gene expression changes that may occur during this part of the fungal life cycle. The objective of this work was to analyze gene transcription during peridiole and basidiospore development, while focusing specifically on cell cycle progression and lipid metabolism.

**Results:**

Throughout different developmental stages of the peridioles we analyzed, 737 genes were regulated between adjacent compartments (>5 fold, FDR-corrected *p*-value < 0.05) corresponding to 3.49% of the genes present in the *P. microcarpus* genome. We identified three clusters among the regulated genes which showed differential expression between the peridiole developmental stages and the basidiospores. During peridiole development, transcripts for proteins involved in cellular processes, signaling, and information storage were detected, notably those for coding transcription factors, DNA polymerase subunits, DNA repair proteins, and genes involved in chromatin structure. For both internal embedded basidiospores (hereto referred to as “Internal spores”, IS) and external free basidiospores (hereto referred to as “Free spores”, FS), upregulated transcripts were found to involve primary metabolism, particularly fatty acid metabolism (FA). High expression of transcripts related to β-oxidation and the glyoxylate shunt indicated that fatty acids served as a major carbon source for basidiosporogenesis.

**Conclusion:**

Our results show that basidiocarp formation in *P. microcarpus* involves a complex array of genes that are regulated throughout peridiole development. We identified waves of transcripts with coordinated regulation and identified transcription factors which may play a role in this regulation. This is the first work to describe gene expression patterns during basidiocarp formation in an ectomycorrhizal gasteromycete fungus and sheds light on genes that may play important roles in the developmental process.

**Electronic supplementary material:**

The online version of this article (doi:10.1186/s12864-017-3545-5) contains supplementary material, which is available to authorized users.

## Background

The basidiomycete fungus *Pisolithus microcarpus* (Coker & Mass.) Cunn. is an ectomycorrhizal mutualistic symbiont able to interact with economically important plants for the forestry sector, such as eucalyptus and acacias [1]. *P. microcarpus* has a worldwide distribution and forms ectomycorrhizal associations with eucalypts and acacias growing in native Australian forests (Western Australia, New South Wales, and Queensland) and exotic forest plantations throughout the world (in Brazil, China, Morocco, Portugal, Senegal, and South Africa) [2]. Phylogeographic studies have shown that *P. microcarpus* first originated in Australia and was subsequently introduced to other locations by human activities [2, 3]. Recent sequencing of the *P. microcarpus* genome [4], the fact that it is easy to handle and remains stable in experimental systems, and its broad geographical distribution make it a model fungus for ecological, physiological, and genetical studies of ectomycorrhizal associations.

**Fig. 1:**
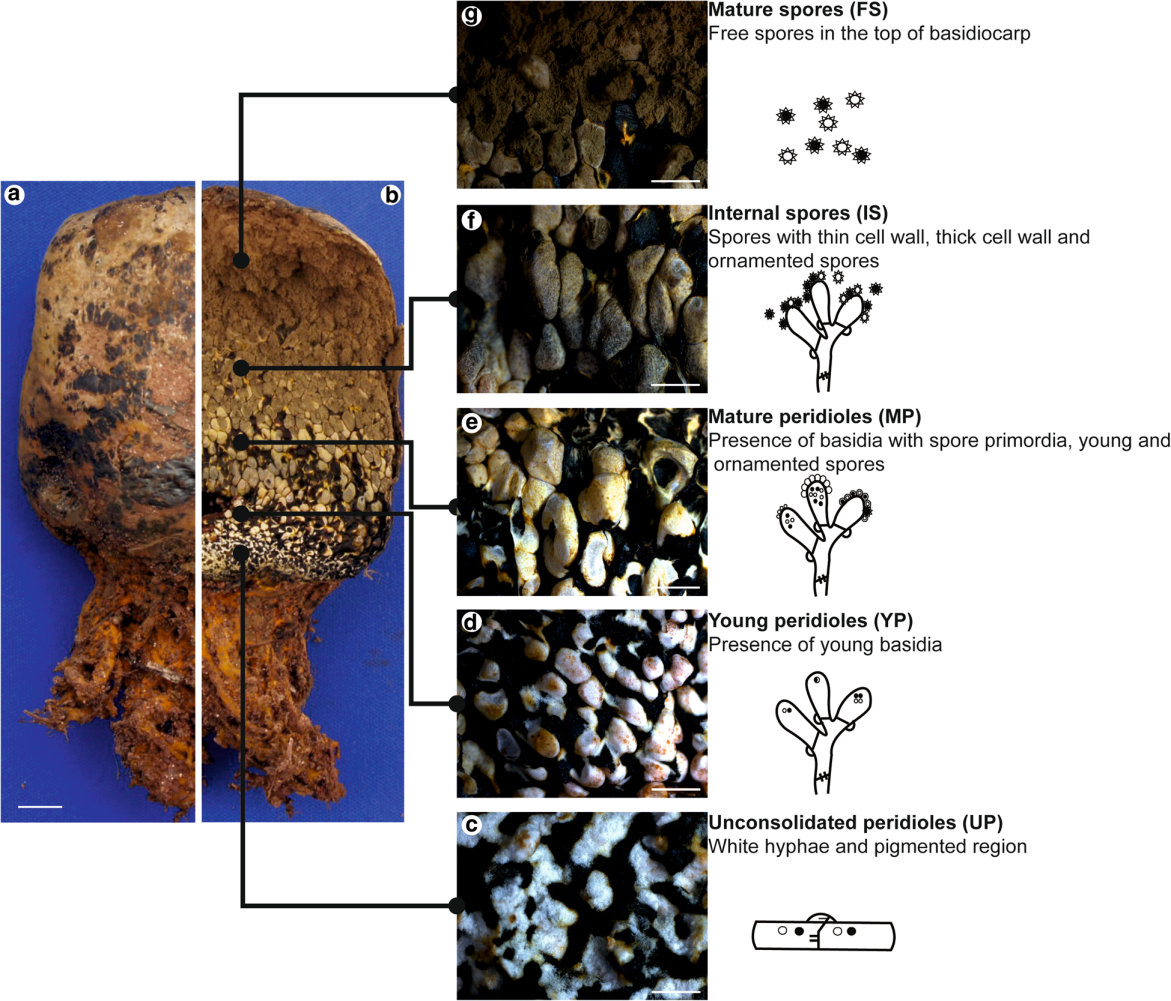
*Pisolithus microcarpus* basidiocarp. General view of a *P. microcarpus* basidiocarp **a** closed and **b** manually opened, showing the five stages of peridiole development described by Campos and Costa (2010) **c** Unconsolidated peridioles (UP), **d** Young peridioles (YP), **e** Mature peridioles (MP), **f** Internal spores (IS), and **g** Free spores (FS). On the right, drawings of microscopical features and characteristics found in the *P. microcarpus* basidiocarp. The scale bar represents 1 cm for the basidiocarps (**a**-**b**) and 0.5 mm for the compartments (**c**-**g**)

Fungi classified in the phylum Basidiomycota form reproductive structures or fruiting bodies called basidiocarps (mushrooms). The production of spores in closed basidiocarps is a common trait of the Sclerodermataceae, a family which includes *P. microcarpus*. The so-called gasteromycetation, the development of hypogeous fruiting bodies from mushroom-shaped fungi is a striking change in fruiting body morphology during evolution. It was explained by secotioid intermediary shapes [5, 6] and it has been suggested to be irreversible since it includes the loss of ballistospory [5, 7]. The *Pisolithus* spp. basidiocarps show wide variation in morphology, including stipe size, shape, length, peridium features, and type and color of spore masses produced inside the fruit body [8–10]. For *P. microcarpus*, the main morphological features of basidiospores and basidiosporogenesis are well known. The fungus produces a complex basidiocarp which serves as a structure within which the peridium (or “nest”) contains small capsules known as peridioles (or “eggs”) in various developmental stages. Enclosed in these peridioles are immature basidiospores and an upper layer of free basidiospores ready for dispersion upon rupture of the basidiocarps cellular cortical layer known as the pellis [11, 12] (Fig. 1a, b). At the base of the basidiocarp, the unconsolidated peridioles are characterized by the presence of white, flattened hyphae densely packed and surrounded by a dark brown pigmented gelatinous matrix containing loosely arranged hyphae (Fig. 1c). The young peridioles are surrounded by the same pigmented matrix and contain only young basidia without spore primordia (Fig. 1d). Mature peridioles are fully developed and contain basidia with spores at different developmental stages (Fig. 1e). Internal spores correspond to those inside partially ruptured peridioles in the upper portion of a closed basidiocarp (Fig. 1f). Finally, free spores are the mature spores that collect at the upper portion of the basidiocarp from fully ruptured peridioles (Fig. 1g) [11].

The large number of basidiospores produced inside the fungal basidiocarp readily facilitates nursery inoculation of eucalyptus seedlings which would allow *P. microcarpus* to be applied to forest production [13]. However, controlled mycorrhization is still limited due, in part, to the low germination rate of the fungal basidiospores and variability in the capacity of homokaryotic mycelia to form ectomycorrhizas. Furthermore, the constitution of lipid reserves and fatty acid distribution during *P. microcarpus* basidiosporogenesis is also known and the evidence that these contents are important for basidiosporogenesis, as well as their distribution throughout the development of the fungal basidiocarp [14]. Despite progress that has been made thus far towards understanding basidiosporogenesis and the distribution of reserve compounds in *P. microcarpus* basidiospores, relatively little is known about the main factors involved in this crucial activation stage of the fungal life cycle [11, 12, 14].

RNA sequencing (RNA-seq) has been used for the transcriptomic analyses of fruiting body development in model fungi such as *Neurospora* sp., *Coprinopsis cinerea*, *Schizophyllum commune*, *Fusarium* sp. and for other fungi with important nutritional and medicinal properties, such as *Cordycepsis militaris*, *Agrocybe aegerita*, *Antrodia cinnamomea*, *Auricularia polytricha*, *Ophiocordyceps sinensis*, *Ganoderma lucidum*, *Tuber melanosporum*, *Agaricus bisporus* and others [15–26]. These works have reported the differential expression of gene families that are regulated during sexual reproduction, signal transduction, defense, secondary metabolism, including volatile organic compounds (VOCs), lipid metabolism, cell cycle, and mating, and a large number of hypothetical proteins have been described. The most common method used to analyze basidiocarp development were done by comparing several stages of development with those for the fungal mycelium [15, 27–30]. Unlike *P. microcarpus*, many ascomycetes and several other studied basidiomycetes have the advantage to form sporocarps in vitro, effectively extending the time frame for producing biological material for transcriptome analyses for each developmental stage. On the other hand, the particular structure of *P. microcarpus* basidiocarp makes it possible to study the different developmental stages of peridioles and basidiospores within a single basidiocarp [11, 31], and effectively facilitate histochemistry, basidiosporogenenesis, and gene expression studies of peridiole development [11, 12, 14].

These transcriptome data is essential for a better understanding of the genes involved in basidiosporogenenis during *P. microcarpus* basidiocarp formation and for determining factors that affect fatty acid metabolism in the basidiocarps and basidiospores. Overall, transcriptome information will contribute to better characterize the different developmental stages of the basidiospores, from cell individualization to maturation and germination, shedding light on the degree of preparedness and the biochemical activities required for subsequent basidiospore germination.

In this work, transcriptome analyses of the stages of peridiole development in *P. microcarpus* basidiocarp [11] were performed using RNA-seq. We present here the most significant changes in gene expression and reveal major metabolic and physiological changes associated with basidiocarp development in *P. microcarpus*. Our analysis of differentially expressed genes (DEGs) focused on those that may contribute to processes responsible for basidiosporogenesis and mobilization of reserve compounds.

## Results

### Gene expression during peridiole development in *Pisolithus microcarpus* basidiocarps

Illumina-RNA sequencing was used to study gene expression in five developmentally different compartments within the *P. microcarpus* basidiocarps, including three peridiole stages as well as internal and free basidiospores (see Additional file 1: Table S1). For about 68% of the predicted 21,064 *P. microcarpus* gene models, transcripts could be identified and were considered as expressed. The majority of transcripts (13,832) were found expressed in all compartments. For those remaining (6443), transcripts could not be found in any of the developmental stages analyzed (see Additional file 2: Figure S1). The number of transcripts without detectable reads is similar for free-living mycelium from *P. microcarpus* 411 (about 6500; data not shown)*,* the sequenced strain and used here as reference genome, indicating that this result is not due to sequence polymorphism. These genes could be either expressed in specific situations or tissues or could represent pseudogenes or misannotations. About 15% of these undetectable transcripts corresponded to genes with predicted functions, such as cellular processes and signaling (4.8%), information storage and processing (5.16%), metabolism (2.12%) while the majority showed no similarity to known domains or functions.

**Fig. 2:**
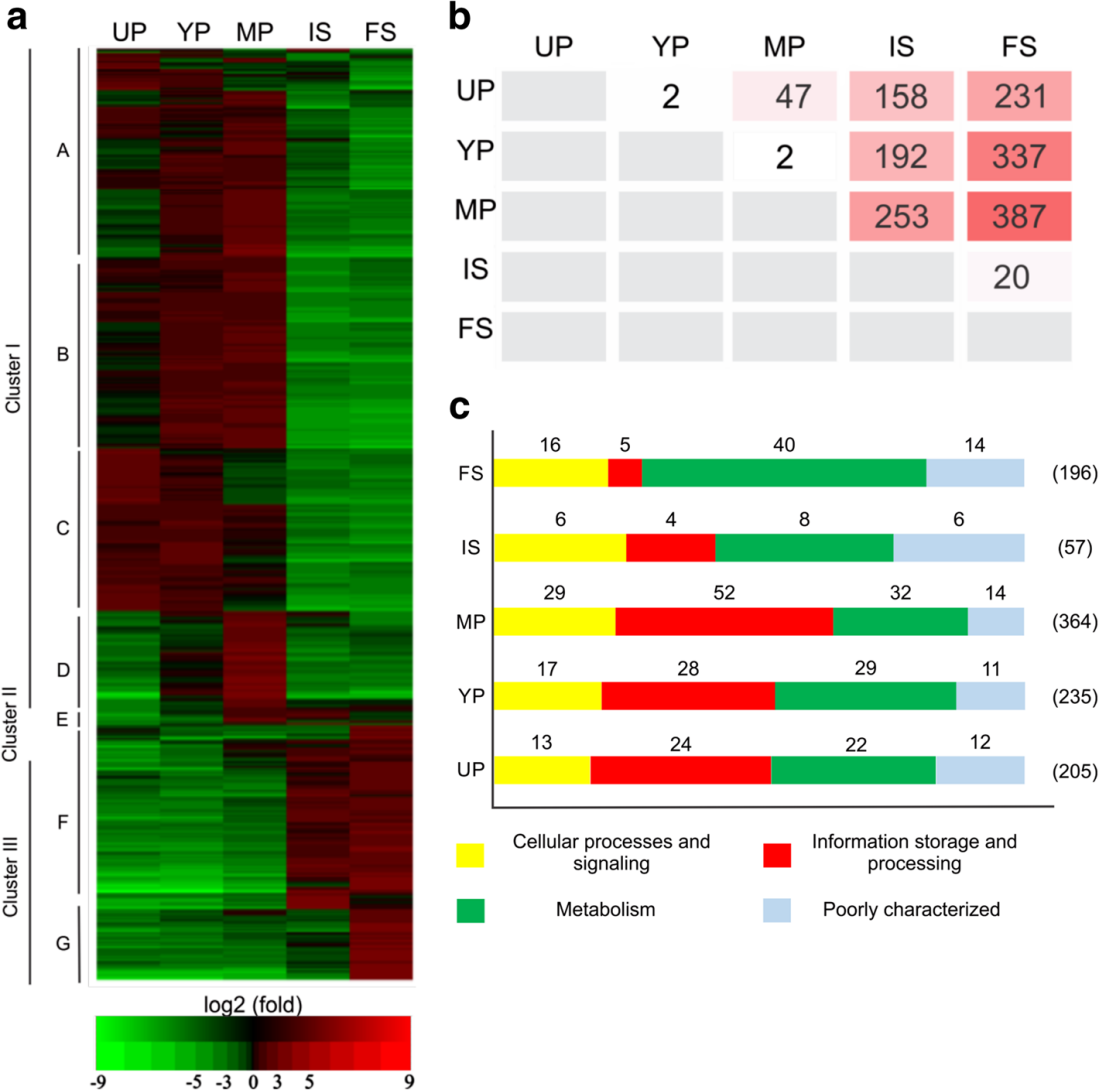
Genes regulated in the five *P. microcarpus* basidiocarp compartments. **a** Hierarchical clustering of 737 transcripts significantly regulated (>5fold, *p*-value < 0.05) between the five compartments. Unconsolidated peridioles (UP), Young peridioles (YP), Mature peridioles (MP), Internal spores (IS) and Free spores (FS). Over-represented (*red*) or under-represented (*green*) transcripts are shown as log_2_ fold changes relative to the mean expression level measured across all five compartments. Letters to the left indicate clusters (see Additional file 4 for data). EPCLUST software was used for the hierarchical clustering **b** Number of regulated genes between basidiocarp compartments identified by pairwise comparison; the color intensity change with the number of genes **c** Functional classification of regulated transcripts using KOG groups. The numbers in parentheses represents the total number of up-regulated genes in each compartment

More than 2500 genes were significantly regulated during peridiole development (FDR *p*-value < 0.05) (see Additional file 3: Figure S2). A total of 737 genes, corresponding to 3.49% of the *P. microcarpus* genes, were differentially regulated more then five-fold in at least one of the five analyzed developmental stages (pairwise comparison, FDR-corrected *p*-value < 0.05) (Fig. 2, Additional file 4: Table S2). A hierarchical clustering of these genes revealed three main clusters (I, II and III) (Fig. 2a). Cluster I corresponds to transcripts up-regulated in one or more of the peridiole compartments (unconsolidated, young or mature peridioles). The smaller cluster II represents transcripts with higher concentrations during the transition from mature peridioles to the basidiospore compartments, while cluster III contains genes up-regulated in basidiospores. Cluster I can be divided in four sub-clusters (A, B, C and D) containing genes highly up-regulated in unconsolidated and/or young and/or mature peridioles and cluster III in two sub-cluster (F and G) (Additional file 4: Table S2). Fig. 2b shows the number of genes regulated (>5fold, FDR *p*-value < 0.05) between the different compartments. The greatest difference was found between mature peridioles and free spores, with 387 differentially expressed transcripts. The EuKaryotic Orthologous Groups (KOG groups) functional classification of the induced transcripts for each compartment (note that the same transcript can be regulated in several compartments) revealed a predominance of information, storage and processing-related transcripts in the peridiole compartments while metabolism-related transcripts were dominant in basidiospore compartments (Fig. 2c).

Table 1 shows the percentage of specific *P. microcarpus* up-regulated transcripts in each compartment compared to the number of specific genes in its genome. Between 15 and 32% of the differentially expressed transcripts are *P. microcarpus* - specific compared to about 25% in the *P. microcarpus* genome. Both the differentially expressed genes in young and mature peridioles were significantly enriched for *P. microcarpus* specific genes (Fischer exact test; *P* < 0.05).

**Table 1:** Percentage of *Pisolithus microcarpus* specific genes amongst the regulated transcripts between compartments^a^

Compartment		Pismi specific^b^ %
Peridioles	Unconsolidated	26.5
	Young	**31.1**
	Mature	**31.9**
Spores	Internal	17.5
	Free	14.8
*Pisolithus microcarpus* genome		24.7

^a^Values in bold: enriched (Fisher exact test; *P* value < 0.05)

^b^A Markov cluster algorithm of predicted proteins from 49 fungal genomes was used. If a cluster contained only predicted proteins from *P. microcarpus* (Pismi), these proteins were considered as Pismi-specific

**Fig. 3:**
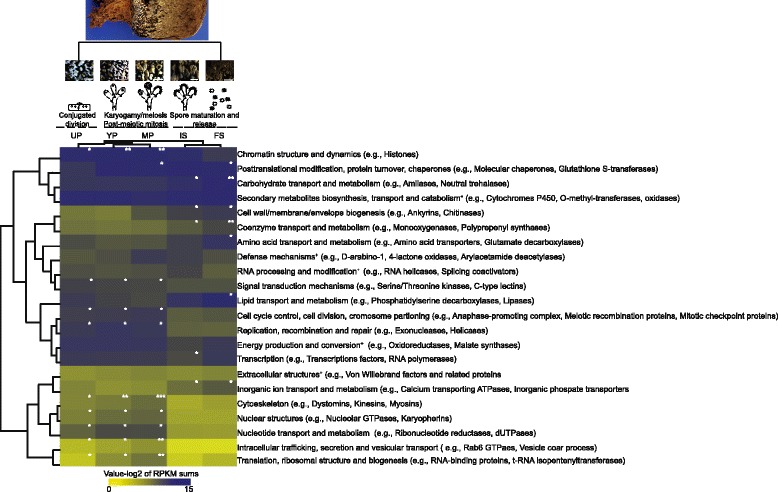
Sum of expression for the regulated transcripts by KOG classes and for each compartment. A double hierarchical clustering by using GENESIS software is shown. Each row represents a KOG class and the expression values are colored in *yellow* (low expression) to *blue* (high expression). A *white* asterisk indicates statistical significance among one of *P. microcarpus* compartments using one-way ANOVA followed by the different multiples pairwise comparison (Tukey, Duncan, Scott-Knot) (*p* < 0.05). On the right, some examples of regulated genes and the corresponding KOG class is given. *Black* plus signal indicates no statistical significance among each compartment was found (*p* < 0.05). Log_2_ values of the RPKM sums were used

To probe deeper into the putative functions of the regulated transcripts in the different compartments, KOG classification was used. Figure 3 shows a double clustering of regulated transcripts (combined by KOG classes; log_2_ sum of RPKM/KOG class) within each compartment. In all peridiole compartments, cell cycle control, cell division and replication-related transcripts were highly expressed compared to transcripts in spores. Up-regulated and highly expressed transcripts for mainly young peridioles, but also mature peridioles, were related to chromatin structure and dynamics as well as to posttranslational modification, protein turnover and catabolism. Cell wall/membrane/envelope biogenesis related transcripts were highly expressed and regulated during the transition from peridioles to spores (mature peridioles, internal and free spores). In the basidiospore compartments, lipid transport and metabolism related transcripts were highly induced, as well as transcripts related to amino acid transport and metabolism. Transcripts for posttranslational modification, protein turnover and catabolism, carbohydrate metabolism, secondary metabolism, energy production and transcription-related transcripts showed to be highly expressed and regulated in all compartments. In general, genes showed similar expression in the peridiole stages (Fig. 3) often with a peak of expression occurring in mature peridioles compared to young and unconsolidated peridioles. The same was true for internal and free spores.

### Differences in gene expression between the basidiocarp compartments

Within unconsolidated peridioles at the bottom of the basidiocarp, 205 transcripts were significantly more abundant than in the other compartments (>5 fold, FDR-corrected *p*-value < 0.05) (Fig. 2c). These genes included many transcription factors with homeobox domain (HOX), high mobility group box (HMG box) and heat shock factor (HSF)-type as well as transcripts involved mainly in replication, transcription and translation (RNA polymerase II subunits, ATP-dependent DNA helicase and helicases). Chitinases, neutral trehalase, α,α-trehalase (GH37) as well as Major Facilitator Superfamily (MFS) and oligopeptide transporters are also induced in this compartment. Following the basidiocarp development, 235 and 364 genes were regulated (>5 fold, FDR-corrected *p*-value < 0.05) (Fig. 2c), respectively in young and mature peridioles. These included transcripts of genes involved in chromatin structure (histones 2A, H3 and H4), meiotic/mitotic processes (DNA replication checkpoint protein, inner centromere protein, serine/threonine, meiotic recombination, mitotic checkpoint serine) and cytoskeleton (microtubule-associated protein). For genes involved in metabolic processes, beta-1,6-N-acetylglucosaminyltransferase and aldehyde dehydrogenase were also up-regulated.

Most of the up-regulated transcripts in internal and free spores (57 and 196 respectively) (Fig. 2c) appeared to be involved in cell metabolism, such as transaldolase, beta-glucocerebrosidase (GH30), N-acetyl-glucosamine-6-phosphate, and beta-glucan synthesis. Additionally, transcripts encoding MFS, calcium, and fucose transporters were only up-regulated in the spore compartment. Furthermore, we observed induction of a family of transcription factors taking place at these stages, such as transcripts coding for HMG and AraC_binding motifs, as well as transcripts involved in signal transduction such as lectins.

### Highly expressed and regulated pathways within the *Pisolithus* basidiocarp

**Fig. 4:**
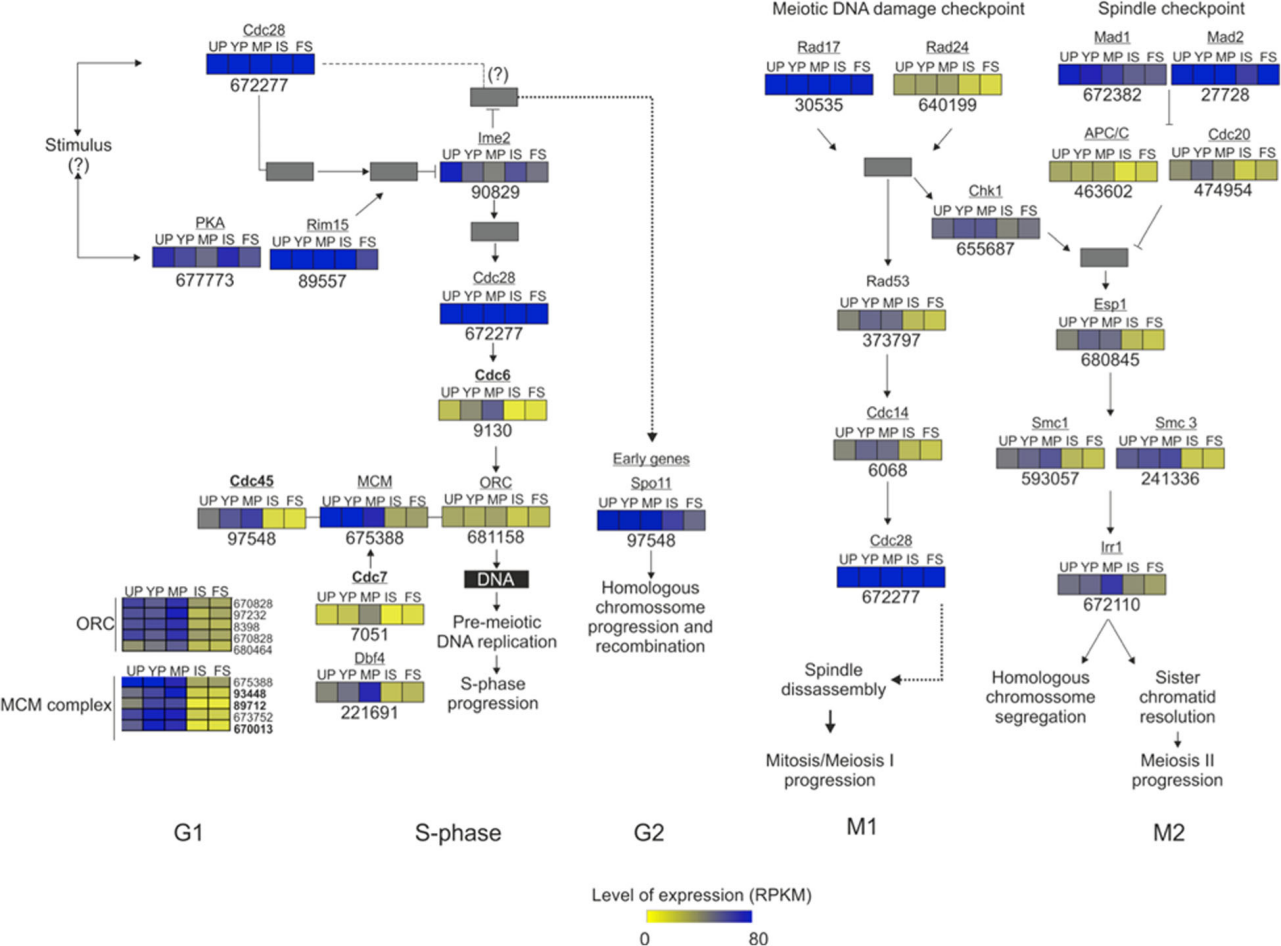
Differential expressed cell cycle related transcripts. For each enzyme, squares represent abundance of transcripts in the different compartments. The numbers under the squares correspond to JGI *P. microcarpus* protein IDs. UP: Unconsolidated peridioles, YP: young peridioles, MP: Mature peridioles, IS: Internal spores, and FS: Free spores

Transcriptome analyses along peridioles and basidiospore compartments revealed two main pathways as highly expressed and up-regulated: cell cycle and lipid metabolism (see Additional file 5: Table S3 and Additional file 6: Table S4). More than 600 transcripts related to cell cycle control, cell division, chromosome partitioning, and cytoskeleton were identified in the genome of *P. microcarpus* [4], out of which 40 transcripts were up-regulated in at least one of the five developmental stages analyzed (see Additional file 7: Figure S3). This set includes transcripts for the anaphase promoting process (APC), essential proteins for meiosis, essential proteins for the S phase, kinesin and myosin. The meiotic pathway in *P. microcarpus* was annotated using the KEGG (Kyoto Gene and Genomes) pathway database and was highly expressed in the peridiole compartments (Fig. 4). The activation of a meiosis/mitosis transcriptional cascade, with sequentially expressed classes of meiosis-specific genes was detected. In G1 and S phases, genes encoding the origin recognition complex (ORC) and the mini-chromosome maintenance complex (MCM) were strongly expressed in all peridiole compartments. An early meiosis-specific gene (*spo11*) showed its highest expression in peridioles compared to the spores. Transcripts for meiosis induction protein kinase (*ime2*) were more abundant in unconsolidated peridioles and internal spores than in the other developmental stages. Following DNA replication, transcripts of genes involved in checkpoint mechanisms for meiosis/mitosis progress, such as *rad17*, *rad24*, *rad53*, *chk1*, and *cdc14* were detected. These transcripts were highly expressed in all *P. microcarpus* compartments, particularly during peridiole development. A set of genes involved in spindle checkpoint process, *mad1* and *mad2*, and a control APC/C function were more highly expressed especially in the peridioles. As for chromosome segregation, transcripts to *cdc7* were up-regulated only in mature peridioles, while *cdc14* and *cdc20* transcripts were accumulated in peridiole compartments. Among the transcripts involved in the structural maintenance of chromosomes, *smc1* and *smc3* also appeared to accumulate in the peridiole compartments.

Given the crucial role lipids play in spore carbon storage [14], we also investigated gene expression related to lipid metabolism in *P. microcarpus* basidiocarp during peridiole development. More than 200 transcripts related to lipid metabolism and transport, as well as to β-oxidation and the glyoxylate cycle proteins are present in the *P. microcarpus* genome (see Additional file 6: Table S4). Thirteen genes involved in lipid metabolism were up-regulated in *P. microcarpus* basidiocarps, including those coding for lysophospholipase, phosphatidylserine decarboxylase, lipases with triacylglycerol lipase activity, a peroxisomal long-chain acyl-CoA transporter and a malate synthase (see Additional file 8: Figure S4). Transcripts involved in fatty acid (FA) biosynthesis, such as FA synthase, acetyl-CoA carboxylase-ACC were highly expressed in spore compartments. In addition, fatty acyl-CoA elongases were highly expressed in peridiole compartments and internal spores with decreased expression in free spores while some transcripts for fatty acid desaturases were abundant in all basidiocarp compartments or had accumulated only in internal spores.

**Fig. 5:**
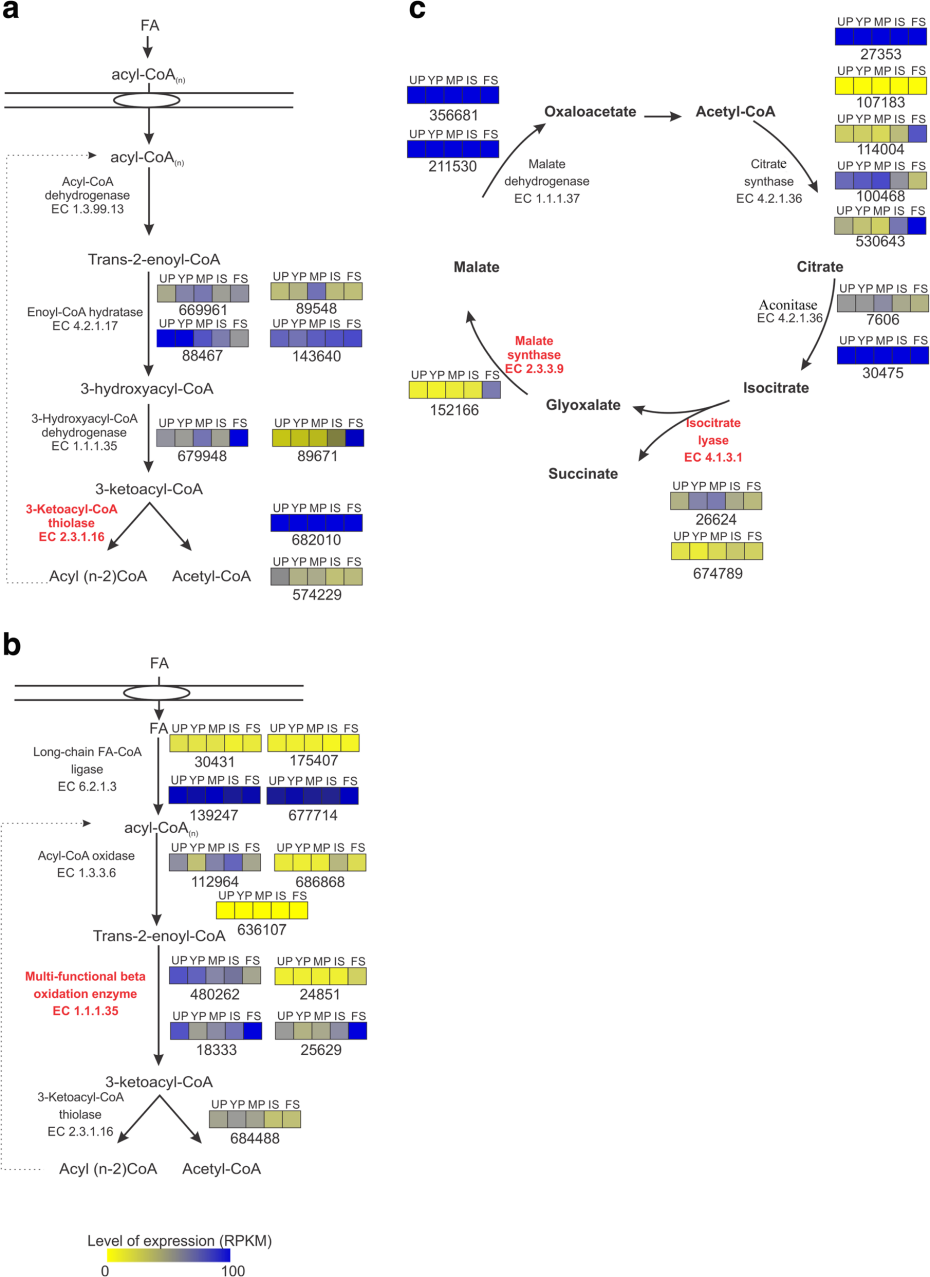
Differential expression of genes coding for lipid metabolism. **a** The β-oxidation pathway in mitochondria, **b** the glyoxylate shunt, and **c** the β-oxidation pathway in peroxisomes. For each enzyme, squares represent abundance of transcripts in the different compartments. The numbers under the squares correspond to JGI *P. microcarpus* protein IDs. UP: Unconsolidated peridioles, YP: young peridioles, MP: Mature peridioles, IS: Internal spores and FS: Free spores

Based on *P. microcarpus* gene annotation and expression analyses, a pathway for β-oxidation in peroxisomes and mitochondria and for the glyoxylate cycle is proposed (Fig. 5). Among these, transcripts to acylcarnitine transferase were found highly expressed in each of the peridiole developmental stages. Accumulated transcripts to acyl-CoA-dehydrogenase, 3-hydroxyacyl-CoA dehydrogenase and β-ketoacyl-coA thiolase were more pronounced in free spores. Long-chain transporters were used for FA import into peroxisomes, some of which were found highly expressed in all developmental stages. The main differences in expression in this pathway were found for the multi-functional enzyme, whose transcripts were up-regulated in free spores.

All gene encoding proteins of the glyoxylate shunt were detected in *P. microcarpus* basidiocarp. Transcripts coding for citrate synthase and aconitase were shown to accumulate during peridiole development while transcripts for key enzymes involved in the glyoxylate cycle such as isocitrate lyase and malate synthase were abundant in free spores. At the end of the glyoxylate shunt, malate dehydrogenase was found highly expressed in all stages, for both internal and free spores.

### Validation of transcriptome data by qRT-PCR

**Fig. 6:**
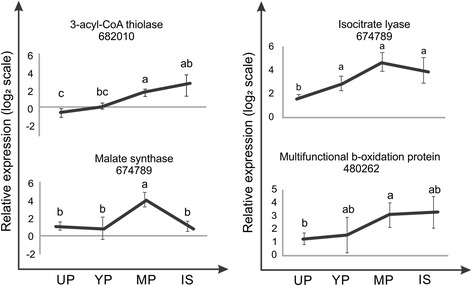
Expression of selected genes in different compartments analysed by qRT-PCR. Relative gene expression (log_2_) in UP: Unconsolidated peridioles, YP: young peridioles, MP: Mature peridioles, and IS: Internal spores. The selected genes were 3-acyl CoA synthase (protein ID 685883), malate synthase (protein ID 152166), isocitrate liase (protein ID 674789), and the multifunction β-oxidation (protein ID 480262) gene. The error bars represent the standard deviation from three independent replicates

A few genes related to lipid metabolism were selected to confirm their expression in the different developmental stages, for unconsolidated, young and mature peridioles and internal spores by qRT-PCR. We tested 3-acyl-CoA thiolase, malate synthase, isocitrate lyase and a peroxisomal multifunctional β-oxidation protein (Fig. 6). All genes showed an increase of expression for the range of unconsolidated to mature peridioles. Transcript concentrations of 3-acyl-CoA thiolase and peroxisomal multifunctional β-oxidation protein were higher in internal spores, while malate synthase and isocitrate lyase transcript accumulation showed expression peaks in mature peridioles. There was also a good correlation between the qRT-PCR and RNA-seq data.

### Expression of basidiocarp development-related genes

To compare the expression and regulation of gene families known to be related to basidiocarp formation in other organisms [32], we investigated genes coding for aquaporins, hydrophobins, C-lectins, expansin-like, haemolysins, laccases and mating-type (see Additional file 9: Figure S5 and Additional file 10: Figure S6). The sum of expression of transcripts coding for aquaporins, haemolysin and for expansin-like proteins showed to be high in peridiole compartments with a subsequent decrease in spore compartments. Hydrophobin genes were among the most highly expressed genes in all compartments of the *P. microcarpus* basidiocarp with a peak of expression in spore compartments. Transcripts for C-lectins were more abundant in unconsolidated peridioles than in other compartments while the transcripts coding to laccases were present in all stages with peaks in mature peridioles and free spores. *P. microcarpus* is a heterothallic fungus with, in addition to the two mating-type genes, three transcripts coding for Ste3-like pheromone receptor, one pheromone activity and two homeodomain genes indicating a possible tetrapolar mating system with multiple mating-type genes for *P. microcarpus*. Only one Ste3-like pheromone receptor was significantly up-regulated within young peridioles. Homeodomain genes were only weakly expressed while mating-type transcripts had accumulated in all peridiole developmental stages, but displayed a peak of expression in internal spores.

**Fig. 7:**
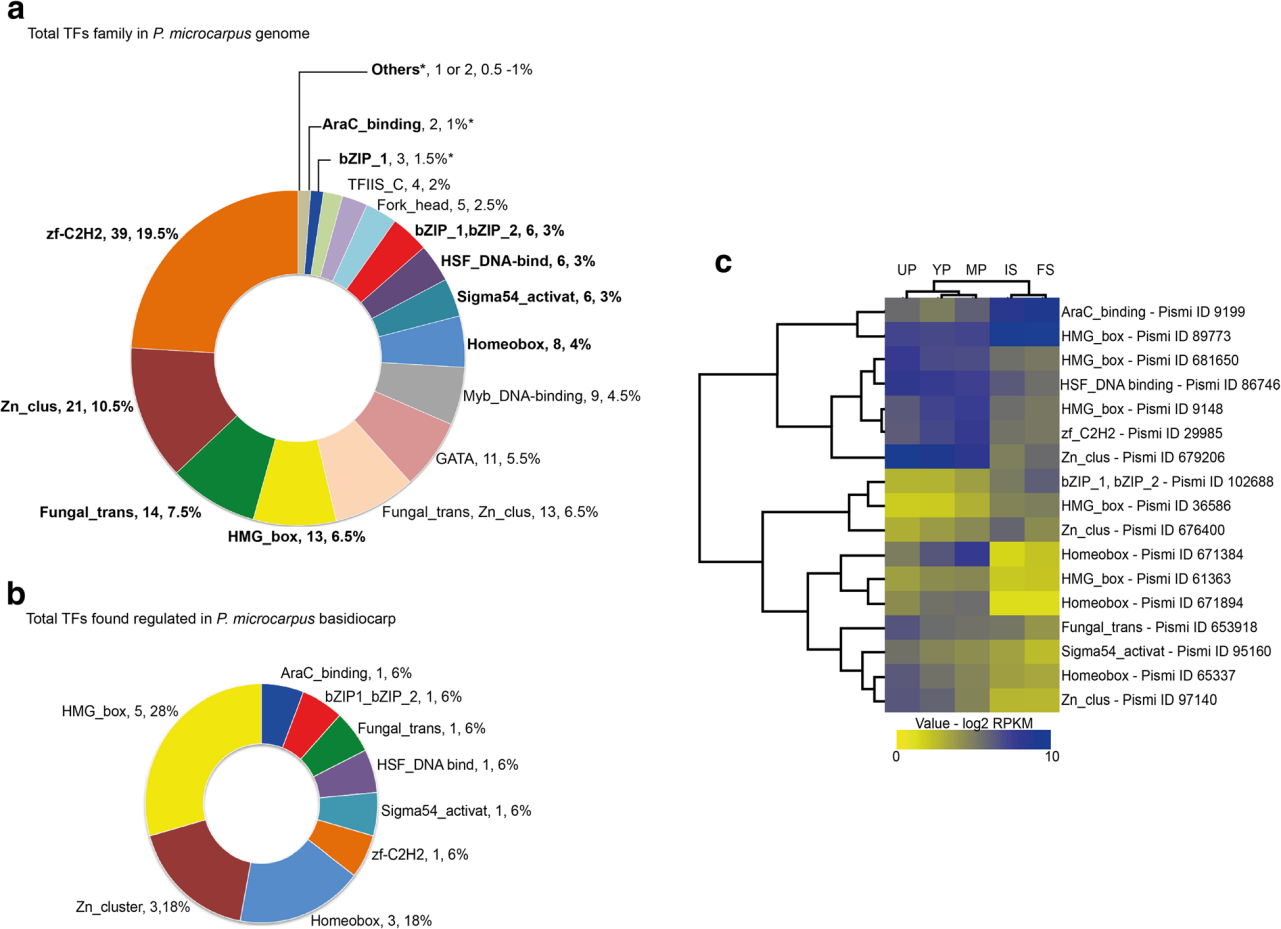
Differential gene expression of transcription factors in the different compartments of *P. microcarpus* basidiocarp. **a** TFs (classified by their domains) present in the *P. microcarpus* genome – TFs family, number of genes, percentage of the total number of TFs (200) found in *P. microcarpus* genome **b** TFs found regulated in *P. microcarpus* basidiocarp compartments, – TFs family, number of regulated genes, percentage of total number of regulated TFs (17) **c** Heat-map with gene expression (log_2_ rpkm). Expression values are colored from *yellow* (low expression) to *blue* (high expression). UP: Unconsolidated peridioles, YP: young peridioles, MP: Mature peridioles, and IS: Internal spores. *Others represents the following TFs family with 1 or 2 domains: ARID, ARID, zf-C5HC2, bZIP_2,bZIP_1, bZIP_2,bZIP_2, CBFB_NFYA, Copper-fist, DDT, DUF592, EnY2, Fungal_trans_2, GCFC, Homeobox_KN, Homeobox, Homeobox_KN, HTH_3, KilA-N, LAG1-DNAbind, RFX_DNA_binding, SART-2, SGT1, SRF-TF, STE,zf-C2H3, TBP, TEA, YABBY, YL1, zf-C2HC6, zf-GRF, zf-MIZ, Zn_clus,Fungal_trans

Due to its crucial role in gene activation, we also investigated the regulation of transcription factor (TFs) gene expression in between the basidiocarp compartments (Fig. 7). The genome of *P. microcarpus* contains 200 genes encoding predicted transcription factors (Fig. 7a). During *P. microcarpus* peridiole development, 17 transcripts coding for TFs were found regulated, corresponding to nine TF families (domains). The most abundant were TFs with HMG_box (5), homeobox and zinc binding (Zn_cluster) domain (3) (Fig. 7b). The heat map shows HMG_box, HSF_DNA binding and Zn_cluster TFs up-regulated in peridioles while TFs involved in specific cellular functions such as nitrogen limitation and fungal specific TFs are up-regulated in both peridiole compartments and internal spores. In contrast, genes coding for TFs with HMG-box (different from those mentioned above), Basic Leucine Zipper (BziP) and AraC domains were more highly expressed in spore compartments (Fig. 7c).

## Discussion

**Fig. 8:**
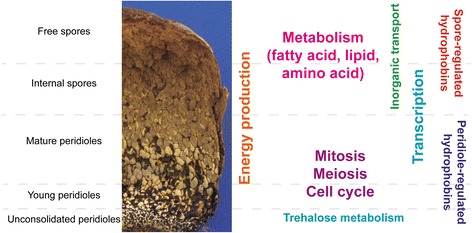
Summary of major events and pathways involved in *P. microcarpus* basidiosporogenesis

This work provides the first glimpse into the transcriptome of a basidiosporogenesis from a gasteromycete fungus with its typical compartments reflecting different developmental stages of peridioles and basidiospores (Fig. 8). These data complete previous studies on basidiosporogenesis [11] and basidiocarp histochemistry [12]. During *P. microcarpus* basidiosporogenesis, in the base of basidiocarp, unconsolidated peridioles consist of an agglomeration of vegetative hyphae that differentiates into basidia. Campos et al. [11, 12] showed that the cell differentiation in this stage requires metabolite precursors such as, lipids, trehalose and glycogen. Consistent with this finding, we observed high levels of expression for genes involved in primary carbon metabolism by regulating oligopeptide transport and trehalose pathways.

In young peridioles, each hyphal cell forms one to several basidia before the final stage where spore primordia are formed inside the mature peridioles. These processes were visible by differential expression of gene families related to signal transduction, replication, chromatin structure, cytoskeleton, and cell cycle control activation. The mature peridioles presented the highest number of regulated genes and in addition, genes coded for clade-specific proteins were significantly enriched in these two compartments. It was not possible, however, to assign functions to these orphan genes with no known domains. This high transcriptional activity, particularly the increase of cell cycle related transcripts, supports the fact that major cell division processes take place in these compartments as Campos and Costa were able to show using fluorescence microscopy and staining with calcofluor white and SYBR Green [11].

Following the differentiation of hyphal cells into basidia [11], basidiospore development takes place with a corresponding increase in deposition of cell wall proteins and further transcriptional changes, especially in catabolic genes, such as beta-1,6-N-acetylglucosaminyltransferases and beta-glucan synthesis, and is consistent with the thick cell wall of *P. microcarpus* spores when they are mature and ready for dispersion.

### Basidiosporogenesis

During *P. microcarpus* basidiosporogenesis, conjugated division, karyogamy, meiosis, post-meiotic mitosis, and nuclear migration events were observed in unconsolidated, young, and mature peridioles [11]. In this work, genes encoding cell cycle proteins were up-regulated and strongly expressed within these compartments. For these processes to occur, cellular growth with an intense increase in replication, transcription, translation, and organelle synthesis is required to prepare for cell division. Several studies have shown that most meiotic genes are conserved in fungi and other eukaryotic organisms, such as plants, protists, and animals [33]. Genes coding for the origin recognition complex (ORC) and the mini-chromosome maintenance complex (MCM) encoding an essential pre-complex formation before the S phase were expressed. This complex binds to DNA strands before the cell enters and starts replication at the S phase. Also, transcripts of cell division cycle proteins (CDC), cyclin-dependent kinases (CDKs), and cyclins were detected. These have been reported to be important for cell cycle regulation mainly in the transition stages of G1/S, G2/M, and M/G1. Expressed in the peridiole compartments, *spo11* has been reported as the main protein responsible for DNA double strand breaks in homologous chromosomes in fungi, such as *C. cinerea* [34]. Our results confirm the occurrence of cell cycle-related activities at the early stages of peridiole development in *P. microcarpus*. For other fungi, such as *Neurospora* sp., transcriptome analyses also revealed correlations between the morphological changes observed during sexual development and cell cycle transcripts [35]. The up-regulation of genes encoding cytoskeleton proteins was observed in all periodiole compartments, especially in mature peridioles, indicating an important activity of organelle transport at these stages. This is consistent with the nuclear migration events in basidia that take place between interphase I and interphase II and that has been shown by fluorescence microscopy [11]. In addition, during spore formation in *C. cinerea*, numerous microtubules are oriented longitudinally in the sterigmata and Golgi vesicles carry carbohydrates to the developing spore and spore wall [36]. In *C. cinerea*, microtubule formation is regulated and necessary for early fruiting events [37]. Overall, our data support the activation of cellular processes that are paramount for basidiosporogenesis, such as nucleus and lipid body migration, cytoskeleton metabolism, and cell wall deposition [11].

### Lipid metabolism

Regulation of a large number of genes coding for proteins involved in primary metabolism, particularly in lipid transport and metabolism, was also observed. During peridiole development, genes coding for phosphatidylserine decarboxylase were consistently expressed, but the expression was approximately seven times higher in free spores than in the other compartments. This enzyme is responsible for converting phosphatidylserine into phophatidylethanolamine, a compound known to accumulate in un-germinated spores of fungi [38, 39]. In addition, the accumulation of triacylglycerol in internal and free spores in *P. microcarpus* possibly indicates that these compounds may be important factors for basidiospore germination, a process currently not well-understood and which may limit *P. microcarpus* inoculation.

Histochemical stains showed that lipid reserves are particularly important in internal and free spores, in that they take up most of the cellular space and push the organelles and the nucleus against the cell wall [14]. Consistent with these stainings, gene transcripts for proteins involved in fatty acids biosynthesis were highly abundant in both the internal and free spores. The presence of unsaturated fatty acids in spores together with the activation of β-oxidation and the glyoxylate cycle during these developmental stages indicate a preparatory stage for the initial mobilization of lipids during basidiospore germination [12, 14, 38]. The up-regulation of genes coding for FA oxidation, such as 3-ketoacyl-CoA thiolase and the multi-functional β-oxidation enzyme protein in mitochondria and peroxisomes showed that in *P. microcarpus* both organelles are involved in fatty acid oxidation. The increase in gene transcripts of the glyoxylate shunt, such as malate synthase in free spores, also confirmed activation of this pathway. This activation of the glyoxylate cycle was also observed in fruiting bodies of other fungi, such as *Tuber borchii*, *Fusarium graminearum*, and *F. verticillioides* [18, 29, 40]. In *C. cinerea*, 38% of the genes encoding proteins involved in cell metabolism were up-regulated at the primordium stage in this fungus [37]. In *A. aegerita*, genes for this pathway were also induced in fruiting bodies [20]. Our results confirm the presence of enzymes for fatty acid elongation during peridiole development and the importance of the glyoxylate shunt for the provision of carbon from lipids during the formation of basidiospores in *P. microcarpus*.

### Other basidiocarp development related gene families

Hydrophobins have previously shown to have high expression levels during fruiting body formation [16, 37]. The peridiole of *P. microcarpus* are embedded in a gelatinous matrix and the presence of hydrophobins in the spore cell walls also exhibited high concentrations [28]. Both findings are consistent with the very high expression of genes coding for hydrophobins within all compartments. In addition, transcripts coding for lectins were accumulated in unconsolidated peridioles suggesting a possible role in defense mechanisms [32, 41]. Transcription factors are essential for many cellular processes and have been identified as regulated in this work, as well as during fruiting body formation in other fungi [16, 42, 43]. During *P. microcarpus* peridiole development, TFs involved in general cellular processes like zinc binding domain-containing (PF10533 and PF00096 for C2H2 type) and HMG-box domains containing TFs were highly expressed in all compartments. Moreover, C2H2-type TFs had the highest expression in primordia and mature fruiting bodies of *S. commune* and *Agaricus bisporus* [16, 32, 44]. HMG-box domain containing TFs are mainly involved in the regulation of DNA-dependent processes such as transcription, replication, and DNA repair, all requiring chromatin conformation changes. This function appeared to correspond with the expression observed in *P. microcarpus* basidiocarp [45]. In all peridioles and enclosed spores, fungus-specific TFs containing the fungal_trans domain (PF04082) were up-regulated in accordance with their known functions in sugar metabolism, gluconeogenesis, respiration and fatty acid catabolism. Fungus-specific TFs were also observed to occur mainly in early stages of fruiting body formation [44]. In addition, TFs with specific cellular functions such as heat-shock stress and nitrogen limitation were present. In spores, TFs with HMG-box (PF00505), BziP (PF00170) and Ara C domain (PF00165) were strongly regulated. These TFs are known to be involved in development, amino acid biosynthesis, nutrient utilization and various stress responses [46]. The distribution and expression of TFs reflect the differential activation of pathways during peridiole and spore development in *P. microcarpus*.

## Conclusions

This is the first work describing the transcriptional landscape within the different compartments of an ectomycorrhizal gasteromycete fungus. We were able to show that genes related to cell cycle regulation and signaling are induced in the peridiole compartments in *P. microcarpus* basidiocarp. The presence of gene coding for proteins involved in lipid metabolism, mainly in fatty acid metabolism, during the last two stages of peridiole development confirm the important role of lipid metabolism during basidiospore production and subsequent germination. These data will contribute to further our understanding the formation and development of fruiting bodies in basidiomycetes, particularly in gasteromycetes, for which most of the genetic, physiological, and morphological processes involved remain poorly characterized.

## Methods

### Biological material and culture conditions

Intact *P. microcarpus* basidiocarps (UFV-VIC 26495) were collected in March 2013 from two eucalypt plantations belonging to Universidade Federal de Viçosa in Viçosa, Minas Gerais State (MG), Brazil (with site location coordinates 20° 49’ 43.4”S, 42° 51’ 52.6”W and 20° 49’ 28.3”S, 42° 51’ 35.8”W). The collected basidiocarps were immediately transported to the laboratory and superficially cleaned with 70% ethanol. The basidiocarps were opened and the gleba representative of the different stages of peridiole development were collected according to [11, 12]. The collected samples were then frozen in liquid nitrogen and stored at -80 °C. A total of three basidiocarps were sampled, each representing one biological replication.

### Total RNA extraction and Illumina transcriptomics

Total RNA of 100 mg of tissue per sample from unconsolidated, young, and mature peridioles, and from internal and free basidiospores, was extracted using the lithium chloride (LiCl) method [47]. Assays for the quantification and integrity check were conducted using an Experion Automated Electrophoresis Station (Bio-Rad, Hercules, CA, USA; See Additional file 11: Figure S7a). Preparation of libraries and 2 x 100 bp Illumina HiSeq mRNA sequencing (RNA-Seq) was performed by Beckman Coulter Genomics following their standard protocol for library construction from 100 ng of total RNA (Danvers, MA, USA). Raw reads were trimmed for low quality (quality score 0.05), Illumina adapters and sequences shorter then 15 nucleotides and aligned to the *P. microcarpus* 441 reference transcripts available at the JGI database http://genome.jgi.doe.gov/Pismi1/Pismi1.home.html [4, 48] using CLC Genomics Workbench v7. Internal transcribed spacer (ITS) sequence from *P. microcarpus 441* was used to fish out ITS reads from FS and MP samples of each of the three basidiocarps and to extract their ITS consensus sequences in order to compare them to the one from *P. microcarpus* 441 (See Additional file 11: Figure S7b).

For read mapping the CLC genomic workbench parameters were the following: minimum length fraction 0.9, minimum similarity fraction 0.8, Mismatch cost = 2, insertion cost = 3, Deletion cost = 3 and the maximum number of hits for a read was set to 10. The unique and total mapped reads number for each transcript were determined and then normalized to RPKM (Reads Per Kilobase of exon model per Million mapped reads). Intact pairs were counted as two, broken pairs as one. Additional file 1: Table S1 summarizes the mapped reads and Additional file 11: Figure S7c shows a principal component analysis (PCA) of all samples.

It should be noted that UP, YP and MP represent dikaryotic tissues with unknown degree of allelic expression while IS and FS are a mix of (post-meiotic) monokaryotic spores of unknown polymorphism.

To identify differentially regulated transcripts in the different fruiting body compartments pairwise comparison by the Baggerley test [49] implemented in CLC Genomic workbench was applied to the data. The Baggerley test compares the proportions of counts in a group of samples against those of another group of samples. The samples are given different weights depending on their sizes (total counts). The weights are obtained by assuming a Beta distribution on the proportions in a group, and estimating these, along with the proportion of a binomial distribution, by the method of moments. The result is a weighted t-type test statistic. In addition Benjamini & Hochberg multiple-hypothesis, testing corrections with False Discovery Rate (FDR) were used. For the present analysis, we focused on transcripts with a more than 5-fold change and a FDR corrected *p*-value <0.05 were used.

For Additional file 2: Figure S1 a gene was considered as expressed with a rpkm >1 and more than 10 reads.

GO, KEGG, KOG and InterPro (IPR) annotations were downloaded from the Joint Genome Institute (JGI) website (http://genome.jgi.doe.gov/Pismi1/Pismi1.home.html) [48]. A Markov cluster algorithm of predicted proteins from 49 fungal genomes [4], including that of *P. microcarpus* (Pismi) (http://genome.jgi.doe.gov/Pismi1/Pismi1.home.html) [48] and of the closely related species *Pisolithus tinctorius* (Pisti) (http://genome.jgi.doe.gov/Pisti1/Pisti1.home.html) [50] was used to identify *P. microcapus* specific proteins. If a protein cluster only contained predicted proteins from *P. microcarpus*, these were considered as Pismi-specific.

For hierarchical clustering EPCLUST (http://www.bioinf.ebc.ee/EP/EP/EPCLUST/) [51] or GENESIS software [52] was used. The complete data set was submitted to GEO GSE93890.

### Identification of pathways and gene families involved in peridiole development in *Pisolithus microcarpus* basidiocarp

A search for homologues of genes involved in cell cycle and lipid metabolism in *P. microcarpus* was conducted using the respective pathways (based on KEGG) from *P. microcarpus* and when the gene was not in the automatic annotation provided by the JGI orthologous genes from *Laccaria bicolor* were used. The annotated sequences in *L. bicolor* were used as query sequences for BLASTP searches for similar proteins in the *P. microcarpus* genome. The BLAST output was sorted and top hits ranked by BLAST scores. The e-value cutoff used to assign homologues was 1e^−5^.

To identify transcription factors we established a list of 330 PFAM domains linked to transcription factor activity, based on the EMBL file available (http://www.transcriptionfactor.org) [53] and pfam2GO terms (Mapping of GO terms to Pfam entries) containing DNA binding transcription factor activity in their GO definition. We then selected the *P. microcarpus* proteins containing these PFAM domains with HMMER package.

### Quantitative real-time PCR (qRT-PCR) validation

Assays for relative quantification were conducted using a Chromo 4TM Detector (MJ Research, Whaltham, USA). The primers were designed using the Primer3 software [54] and AmplyfX (http://crn2m.univ-mrs.fr/pub/amplifx-dist) [55] (see Additional file 12: Table S5). Each qRT-PCR reaction contained one ng of cDNA, 300 of each primer and 2x iQ SYBR Green Supermix (BioRad). All reactions were made in triplicate using three preparations of total RNA from independent biological samples (basidiocarps) and amplified at 94 °C for 3 min, 40x at 94 °C for 15 s and 60 °C for 1 min. Three reference genes, actin, tubulin, and elongation factor were used in this work. The reference genes were selected by using BestKeeper [56]. Tests for amplification efficiency and assay validation were performed as described by [57]. Primers designed for intron regions were used for gDNA contamination control. Relative expression was calculated by MCS-REST and the comparisons were performed using one-way analysis of variance and Wilcoxon test in R program.

## Additional files

Additional file 1: Table S1. Summary of read alignments to the reference transcripts. (XLSX 25 kb)
Additional file 2: Figure S1. Venn diagram showing the number of genes expressed and not expressed in *P. microcarpus* basidiocarp. UP: Unconsolidated peridioles, YP: young peridioles, MP: Mature peridioles, IS: Internal spores, and FS: Free spores. (DOCX 360 kb)
Additional file 3: Figure S2. Number of genes significantly regulated between the different compartments. The number of significantly regulated transcripts (FDR *p*-value < 0.05), with an additional cut-off >2fold as well as with an additional cut-off >5-fold are given for each pairwise comparison. UP: Unconsolidated peridioles, YP: young peridioles, MP: Mature peridioles, IS: Internal spores, and FS: Free spores (DOCX 30 kb)
Additional file 4: Table S2. List of differentially expressed genes between compartments of the *Pisolithus microcarpus* basidiocarp. UP: Unconsolidated peridioles, YP: young peridioles, MP: Mature peridioles, IS: Internal spores, and FS: Free spores. (XLSX 363 kb)
Additional file 5: Table S3. List of genes from cell cycle, cytoskeleton and nuclear structure. UP: Unconsolidated peridioles, YP: young peridioles, MP: Mature peridioles, IS: Internal spores and FS: Free spores. (XLSX 427 kb)
Additional file 6: Table S4. List of genes involved in lipid metabolism. UP: Unconsolidated peridioles, YP: young peridioles, MP: Mature peridioles, IS: Internal spores, and FS: Free spores. (XLSX 126 kb)
Additional file 7: Figure S3. Heat-map of regulated genes coding for cell cycle, cytoskeleton and nuclear structure related proteins in between compartments from *P. microcarpus* basidiocarp. UP: Unconsolidated peridioles, YP: young peridioles, MP: Mature peridioles, IS: Internal spores, and FS: Free spores. (DOCX 253 kb)
Additional file 8: Figure S4. Heat-map of regulated genes involved in lipid metabolism. UP: Unconsolidated peridioles, YP: young peridioles, MP: Mature peridioles, IS: Internal spores, and FS: Free spores. (DOCX 165 kb)
Additional file 9: Figure S5. Changes in gene expression of mating-types genes in *P. microcarpus* basidiocarp. UP: Unconsolidated peridioles, YP: young peridioles, MP: Mature peridioles, IS: Internal spores, and FS: Free spores. (DOCX 463 kb)
Additional file 10: Figure S6. Changes in gene expression of gene families related with basidiocarp formation. (DOCX 124 kb)
Additional file 11: Figure S7. Sample control. A Internal transcribed spacer (ITS) sequences from selected samples compared to the ITS sequence from *Pisolithus microcarpus (Pm)* 441 used as reference. The ITS sequence of Pm441 was used to map reads from selected samples to it, then consensus sequences were extracted and compared to each other using Multalin. B Experion profiles from total RNA used for library construction. C Principal component analysis of rpkm normalised samples (CLC genomic workbench quality control). (PPTX 671 kb)
Additional file 12: Table S5. Primer list used for qPCR. (XLSX 23 kb)

## Abbreviations

APC: Anaphase promoting process
BziP: Basic Leucine Zipper
CDC: Cell division cycle proteins
CDKs: Cyclin-dependent kinases
DEGs: Differentially expressed genes
FA: Fatty acid
FDR: Fold discovery rate
FS: Free spores
GEO: Gene expression omnibus
GO: Gene ontology
HMG box: High mobility group box
HOX: Homeobox domain
HSF: Heat shock factor
IPR: InterPro
IS: Internal spores
ITS: Internal transcribed spacer
JGI: Joint Genome Institute
KEGG: Kyoto Gene and Genomes
KOG: EuKaryotic Orthologous Groups
LiCl: Lithium chloride
MCM: Mini-chromosome maintenance complex
MFS: Major facilitator superfamily
MP: Mature peridioles
NCBI: National Center for Biotechnology Information
ORC: Origin recognition complex
PCA: Principal component analysis
Pismi: *Pisolithus microcarpus*
Pisti: *Pisolithus tinctorius*
qRT-PCR: Quantitative real-time PCR
RNA-seq: RNA sequencing
RPKM: Reads per kilo base per million mapped reads
TF: Transcription factor
UP: Unconsolidated peridioles
VOC: Volatile organic compounds
YP: Young peridioles
Zn_cluster: Zinc binding domain
