# Supplementary material for: The transcriptional landscape of basidiosporogenesis in mature Pisolithus microcarpus basidiocarp
Source: BMC Genomics. 2017 Feb 14;18:157. doi: 10.1186/s12864-017-3545-5 (PMC5310086; doi:10.1186/s12864-017-3545-5)
Supplement: Additional file 2: Figure S1. — Venn diagram showing the number of genes expressed and not expressed in P. microcarpus basidiocarp. UP: Unconsolidated peridioles, YP: young peridioles, MP: Mature peridioles, IS: Internal spores, and FS: Free spores. (DOCX 360 kb) [file 12864_2017_3545_MOESM2_ESM.docx]

**
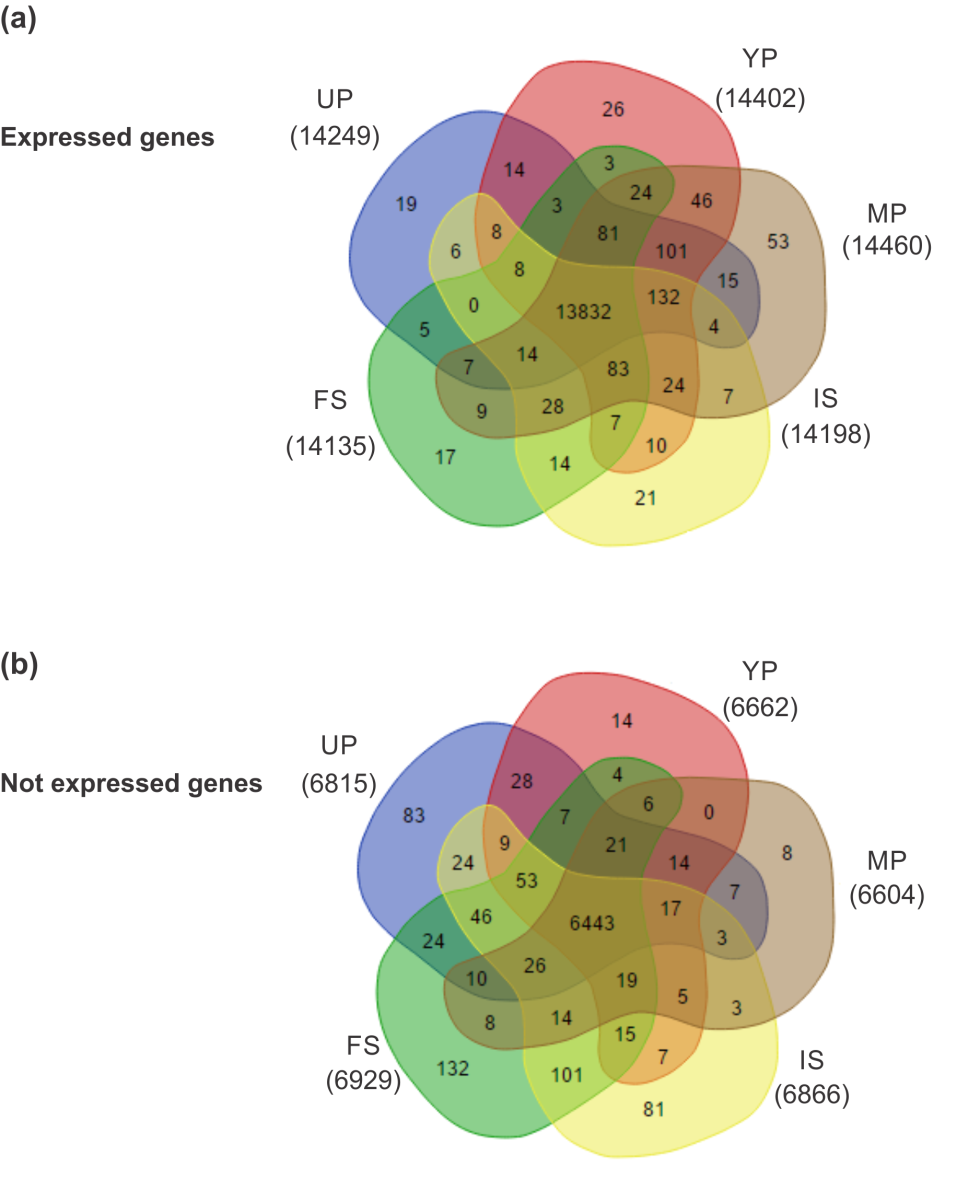
**

**Additional file 2: Figure S1.** Venn diagram showing the number of genes in *P. microcarpus* basidiocarp (a) with expression levels higher than 1 rpkm and (b) the not expressed genes with rpkm between >0 and <1. A minimum of 10 reads was necessary for genes to be considered as expressed. Numbers in parentheses indicate the number of genes expressed or not in each compartment, Unconsolidated peridioles (UP), Young peridioles (YP), Mature peridioles (MP), Internal spores (IS) and Free spores (FS). The Venn diagrams were created using a web tools provided by the Bioinformatics and Systems Biology of Gent, Belgium (<http://bioinformatics.psb.ugent.be/webtools/Venn/>).
