# Supplementary material for: The transcriptional landscape of basidiosporogenesis in mature Pisolithus microcarpus basidiocarp
Source: BMC Genomics. 2017 Feb 14;18:157. doi: 10.1186/s12864-017-3545-5 (PMC5310086; doi:10.1186/s12864-017-3545-5)
Supplement: Additional file 3: Figure S2. — Number of genes significantly regulated between the different compartments. The number of significantly regulated transcripts (FDR p-value < 0.05), with an additional cut-off >2fold as well as with an additional cut-off >5-fold are given for each pairwise comparison. UP: Unconsolidated peridioles, YP: young peridioles, MP: Mature peridioles, IS: Internal spores, and FS: Free spores (DOCX 30 kb) [file 12864_2017_3545_MOESM3_ESM.docx]

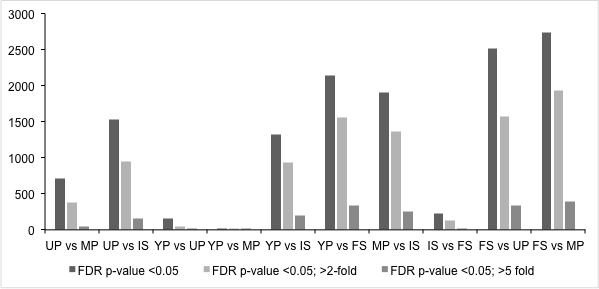


**Additional file 3: Figure S2.** Number of genes significantly regulated between the different compartments. The number of significantly regulated transcripts (FDR p-value<0.05), with an additional cut-off >2fold as well as with an additional cut-off >5-fold are given for each pairwise comparison. UP: Unconsolidated peridioles, YP: young peridioles, MP: Mature peridioles, IS: Internal spores, and FS: Free spores.
