# Supplementary material for: The transcriptional landscape of basidiosporogenesis in mature Pisolithus microcarpus basidiocarp
Source: BMC Genomics. 2017 Feb 14;18:157. doi: 10.1186/s12864-017-3545-5 (PMC5310086; doi:10.1186/s12864-017-3545-5)
Supplement: Additional file 7: Figure S3. — Heat-map of regulated genes coding for cell cycle, cytoskeleton and nuclear structure related proteins in between compartments from P. microcarpus basidiocarp. UP: Unconsolidated peridioles, YP: young peridioles, MP: Mature peridioles, IS: Internal spores, and FS: Free spores. (DOCX 253 kb) [file 12864_2017_3545_MOESM7_ESM.docx]

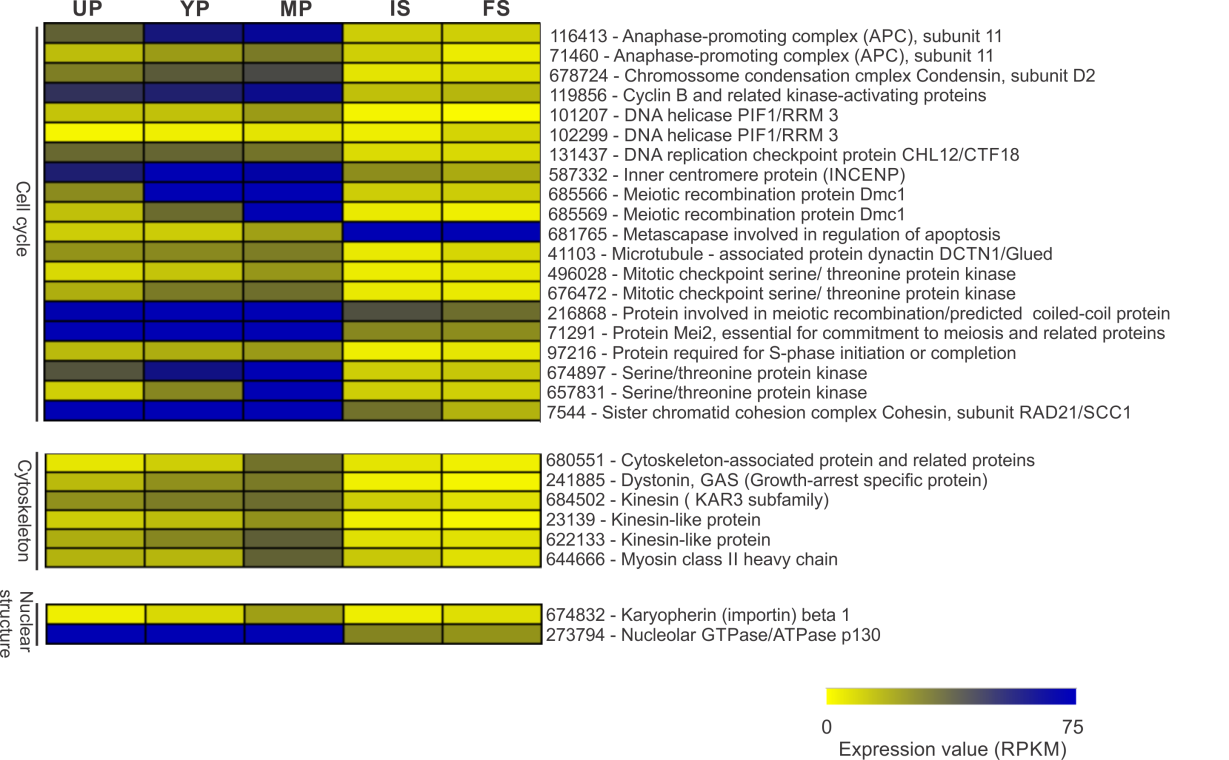


**Additional file 7: Figure S3:** Heat-map of regulated genes corresponding cell cycle, cytoskeleton and nuclear structure during peridioles development in *P. microcarpus* basidiocarp. For each protein, square color represents the abundance of transcripts encoding for genes in unconsolidated peridioles (UP), young peridioles (YP), mature peridioles (MP), internal spores (IS) and free spores (FS) following by protein ID JGI and protein name.
