# Supplementary material for: The transcriptional landscape of basidiosporogenesis in mature Pisolithus microcarpus basidiocarp
Source: BMC Genomics. 2017 Feb 14;18:157. doi: 10.1186/s12864-017-3545-5 (PMC5310086; doi:10.1186/s12864-017-3545-5)
Supplement: Additional file 8: Figure S4. — Heat-map of regulated genes involved in lipid metabolism. UP: Unconsolidated peridioles, YP: young peridioles, MP: Mature peridioles, IS: Internal spores, and FS: Free spores. (DOCX 165 kb) [file 12864_2017_3545_MOESM8_ESM.docx]

**
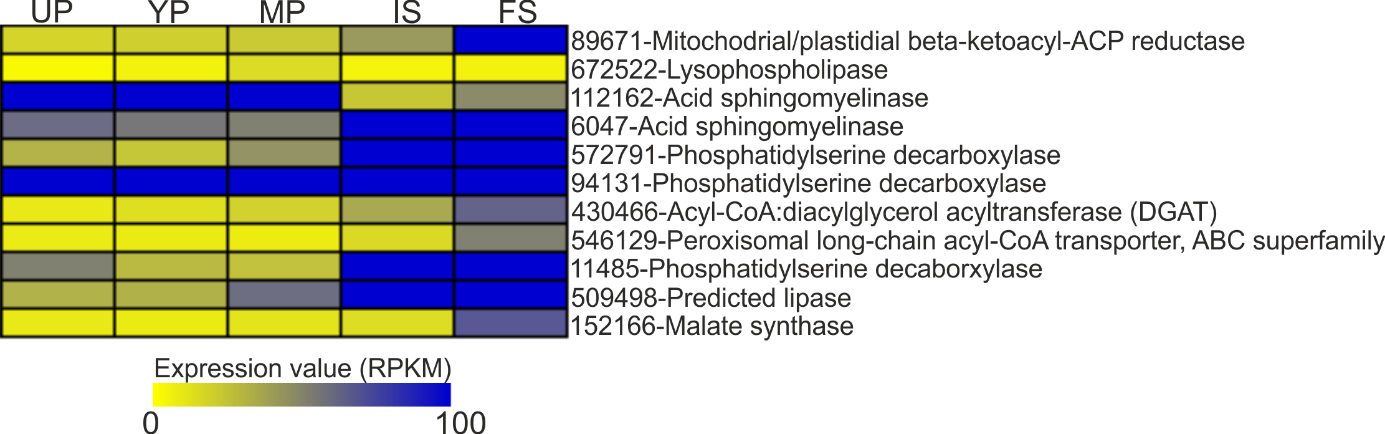
**

**Additional file 8: Figure S4:** Heat-map of regulated genes corresponding lipid metabolism during the development of peridioles. For each protein, square color represents the abundance of transcripts encoding for genes in unconsolidated peridioles (UP), young peridioles (YP), mature peridioles (MP), internal spores (IS) and free spores (FS) following by protein ID JGI and protein name.
