# Supplementary material for: The transcriptional landscape of basidiosporogenesis in mature Pisolithus microcarpus basidiocarp
Source: BMC Genomics. 2017 Feb 14;18:157. doi: 10.1186/s12864-017-3545-5 (PMC5310086; doi:10.1186/s12864-017-3545-5)
Supplement: Additional file 9: Figure S5. — Changes in gene expression of mating-types genes in P. microcarpus basidiocarp. UP: Unconsolidated peridioles, YP: young peridioles, MP: Mature peridioles, IS: Internal spores, and FS: Free spores. (DOCX 463 kb) [file 12864_2017_3545_MOESM9_ESM.docx]

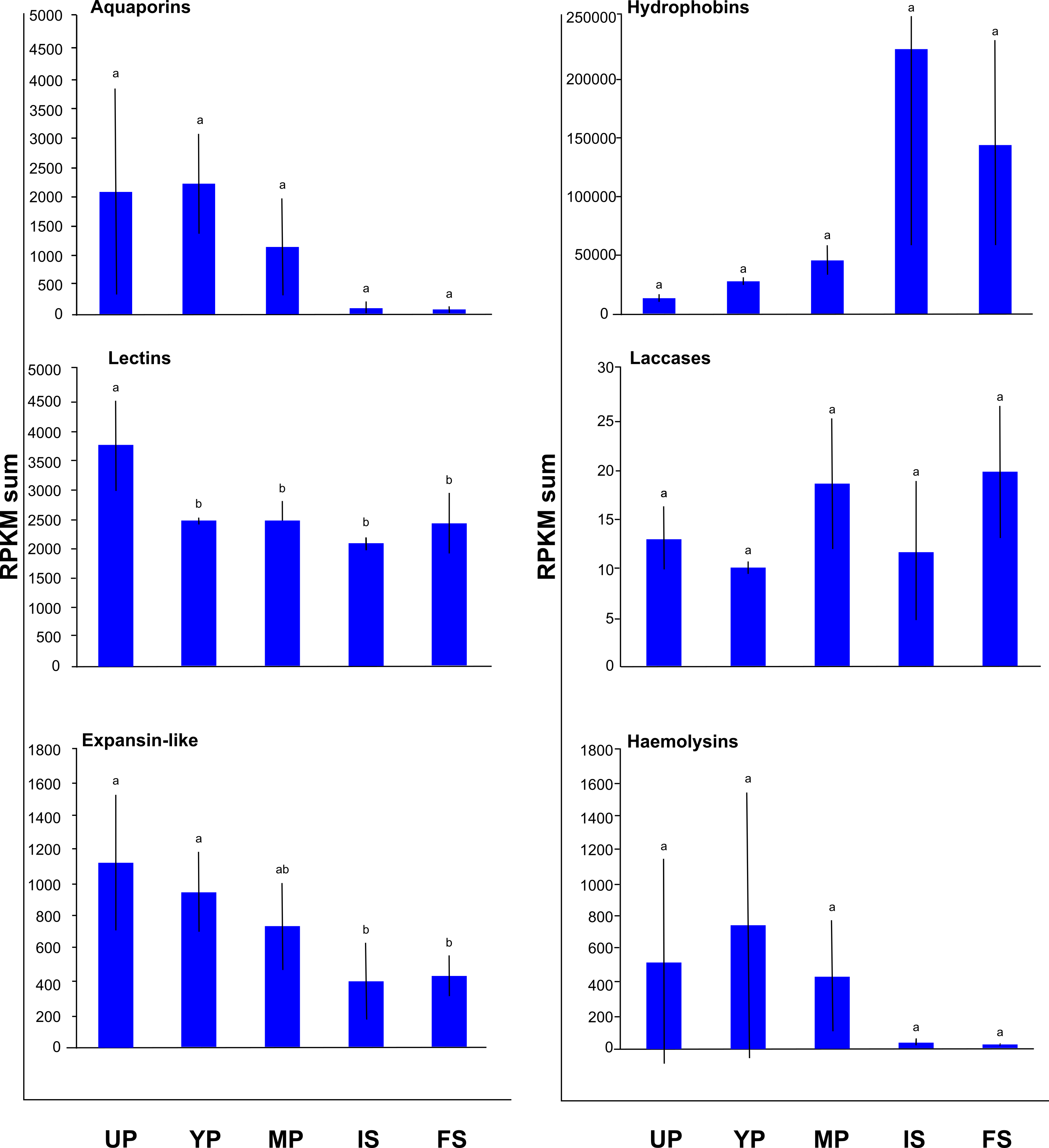


**Additional file 9: Figure S5.** Changes in gene expression of gene families related with basidiocarp formation. The sum of expression (RPKM) of transcripts coding for aquaporins, hydrophobins, lectins, laccases, expansin-like and haemolysin were calculated for each *P. microcarpus* basidiocarp compartment. Lower case letters denote statistical significance using one-way ANOVA followed by the Tukey pairwise comparison (p < 0.05). Error bars are the standard deviations of three biological triplicates. UP: Unconsolidated peridioles, YP: young peridioles, MP: Mature peridioles, IS: Internal spores, and FS: Free spores.
