# Supplementary material for: The transcriptional landscape of basidiosporogenesis in mature Pisolithus microcarpus basidiocarp
Source: BMC Genomics. 2017 Feb 14;18:157. doi: 10.1186/s12864-017-3545-5 (PMC5310086; doi:10.1186/s12864-017-3545-5)
Supplement: Additional file 10: Figure S6. — Changes in gene expression of gene families related with basidiocarp formation. (DOCX 124 kb) [file 12864_2017_3545_MOESM10_ESM.docx]

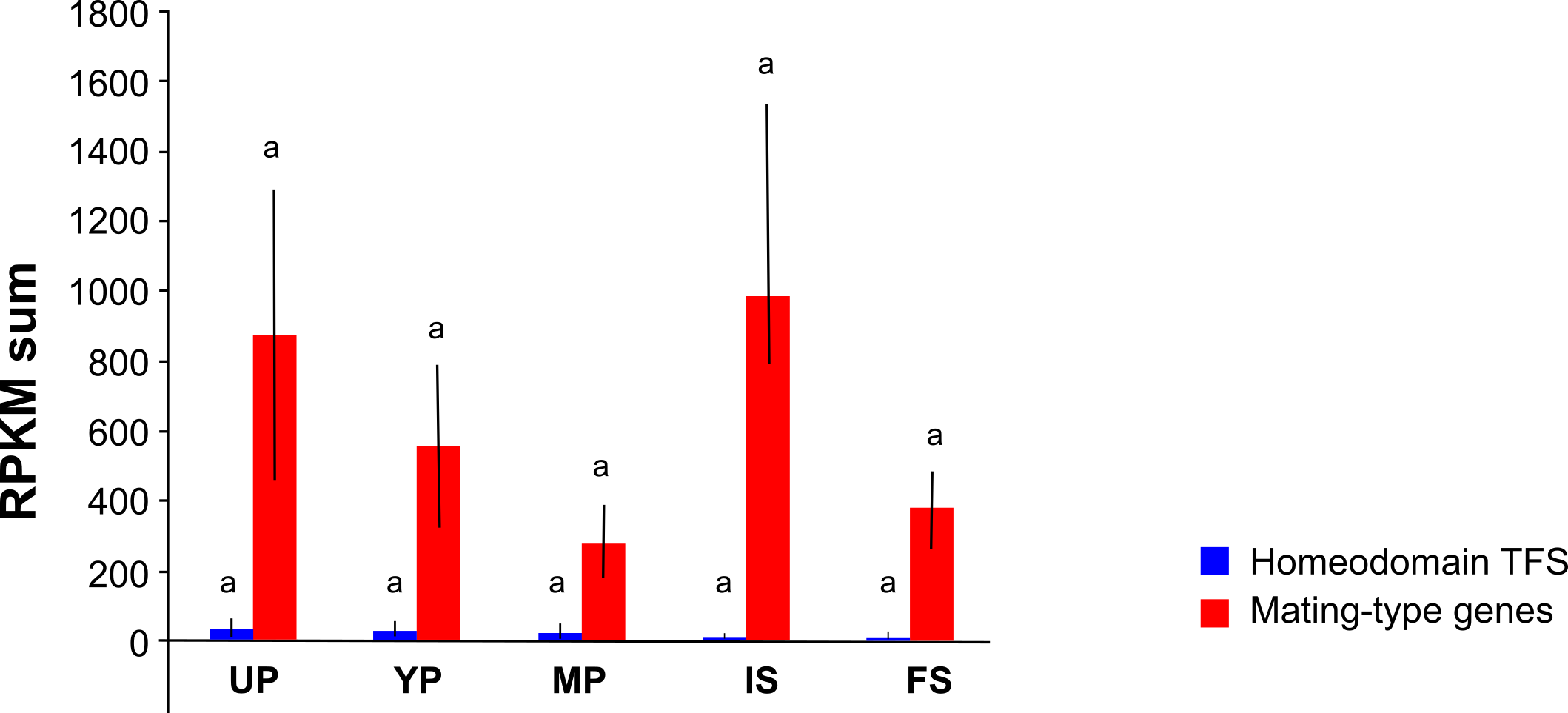


**Additional file 10: Figure S6.** Changes in gene expression of mating-types genes. Sum of expression (RPKM) of genes encoding to homeodomain TFs, receptors and pheromones involved in mating-type processes in each compartment of *P. microcarpus* basidiocarp. Lower case letters denote statistical significance using one-way ANOVA followed by the Tukey pairwise comparison (p < 0.05). Error bars are the standard deviations of three biological triplicates. UP: Unconsolidated peridioles, YP: young peridioles, MP: Mature peridioles, IS: Internal spores, and FS: Free spores.
