# Supplementary material for: The transcriptional landscape of basidiosporogenesis in mature Pisolithus microcarpus basidiocarp
Source: BMC Genomics. 2017 Feb 14;18:157. doi: 10.1186/s12864-017-3545-5 (PMC5310086; doi:10.1186/s12864-017-3545-5)
Supplement: Additional file 11: Figure S7. — Sample control. A Internal transcribed spacer (ITS) sequences from selected samples compared to the ITS sequence from Pisolithus microcarpus (Pm) 441 used as reference. The ITS sequence of Pm441 was used to map reads from selected samples to it, then consensus sequences were extracted and compared to each other using Multalin. B Experion profiles from total RNA used for library construction. C Principal component analysis of rpkm normalised samples (CLC genomic workbench quality control). (PPTX 671 kb) [file 12864_2017_3545_MOESM11_ESM.pptx]

## Slide 1
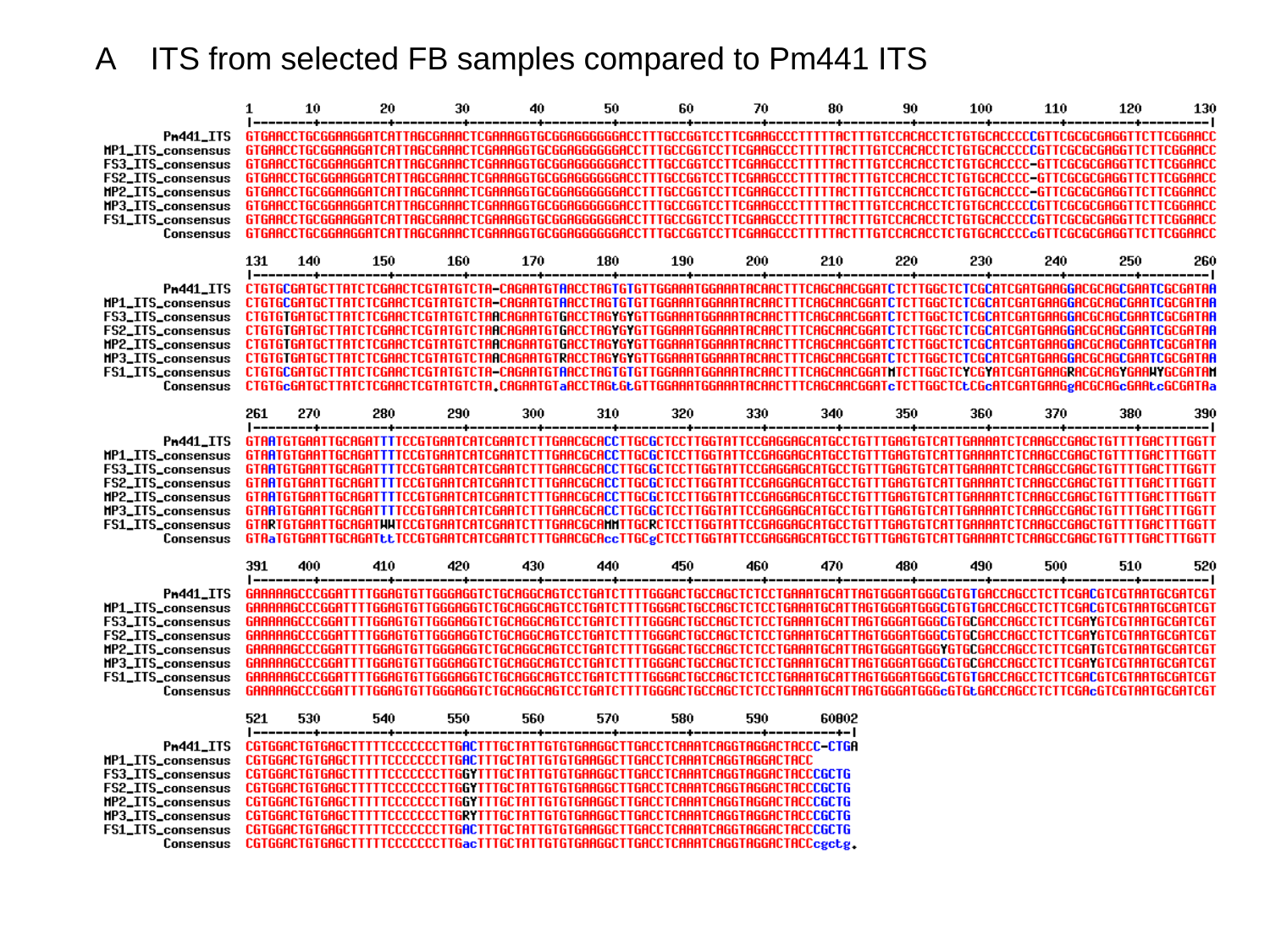

A ITS from selected FB samples compared to Pm441 ITS

## Slide 2
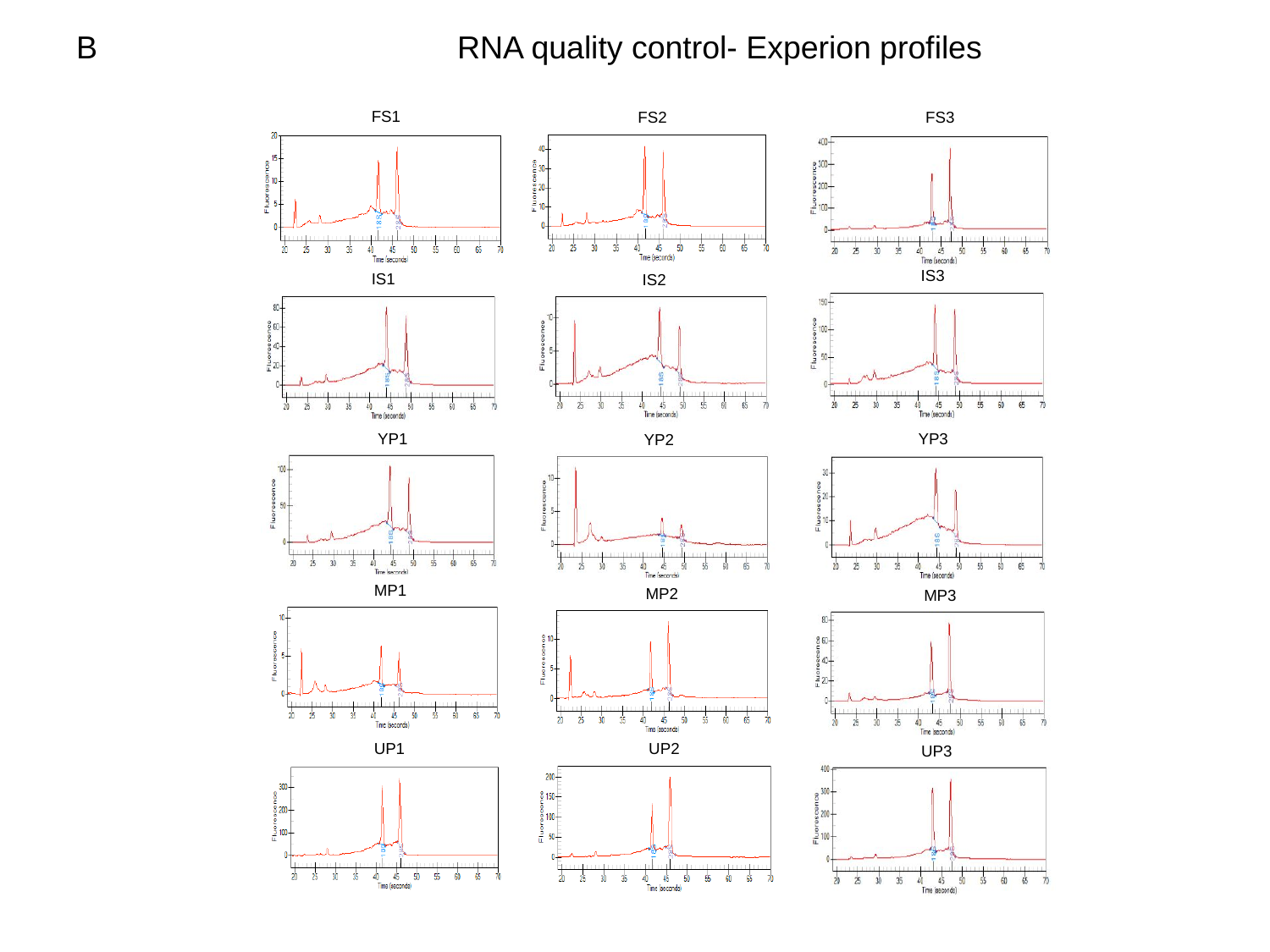

B 			RNA quality control- Experion profiles
FS1
FS2
FS3
IS3
IS1
IS2
YP1
YP3
YP2
MP1
MP2
MP3
UP1
UP2
UP3

## Slide 3
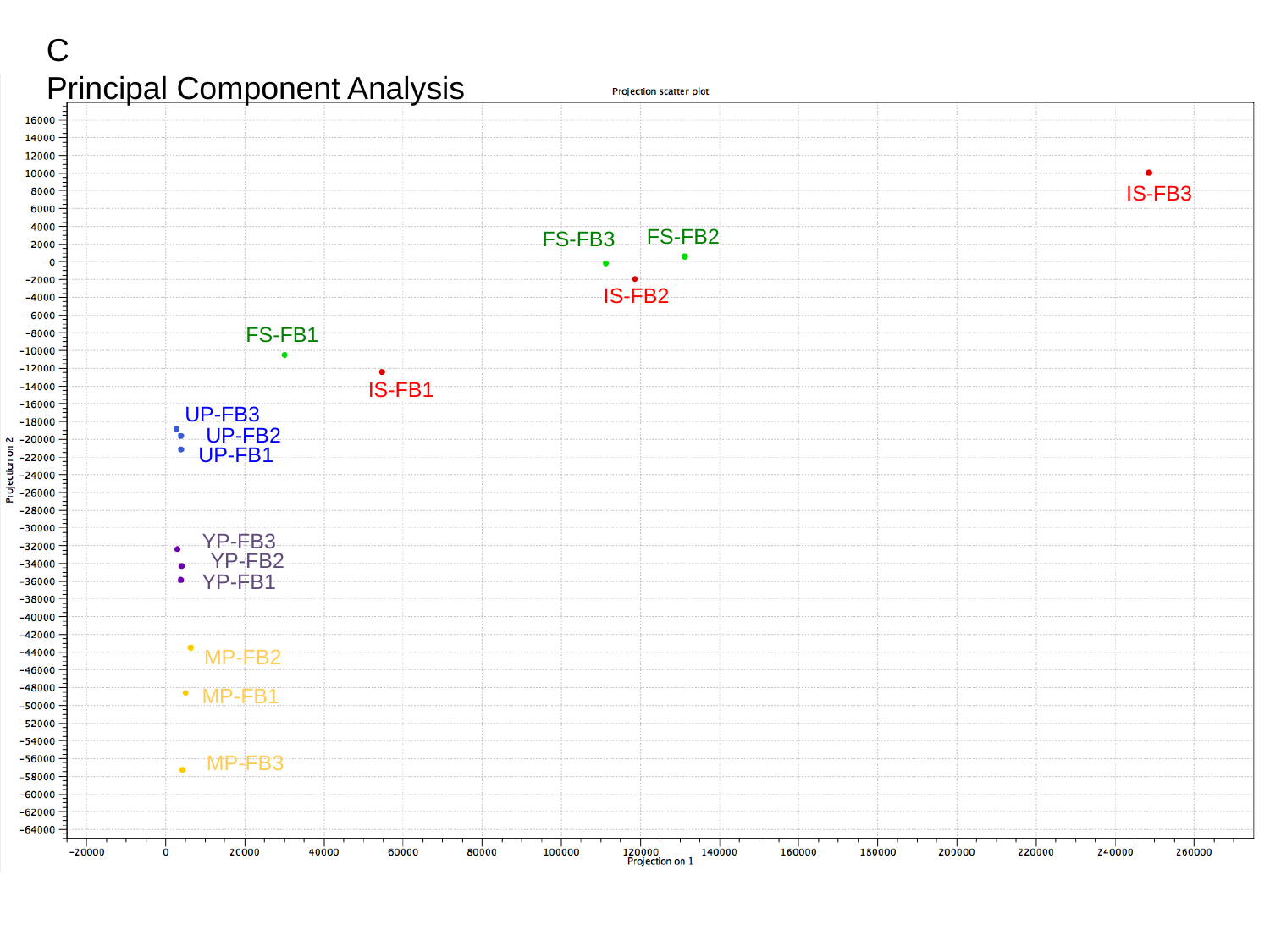

C 						Principal Component Analysis
IS-FB3
FS-FB2
FS-FB3
IS-FB2
FS-FB1
IS-FB1
UP-FB3
UP-FB2
UP-FB1
YP-FB3
YP-FB2
YP-FB1
MP-FB2
MP-FB1
MP-FB3

## Slide 4
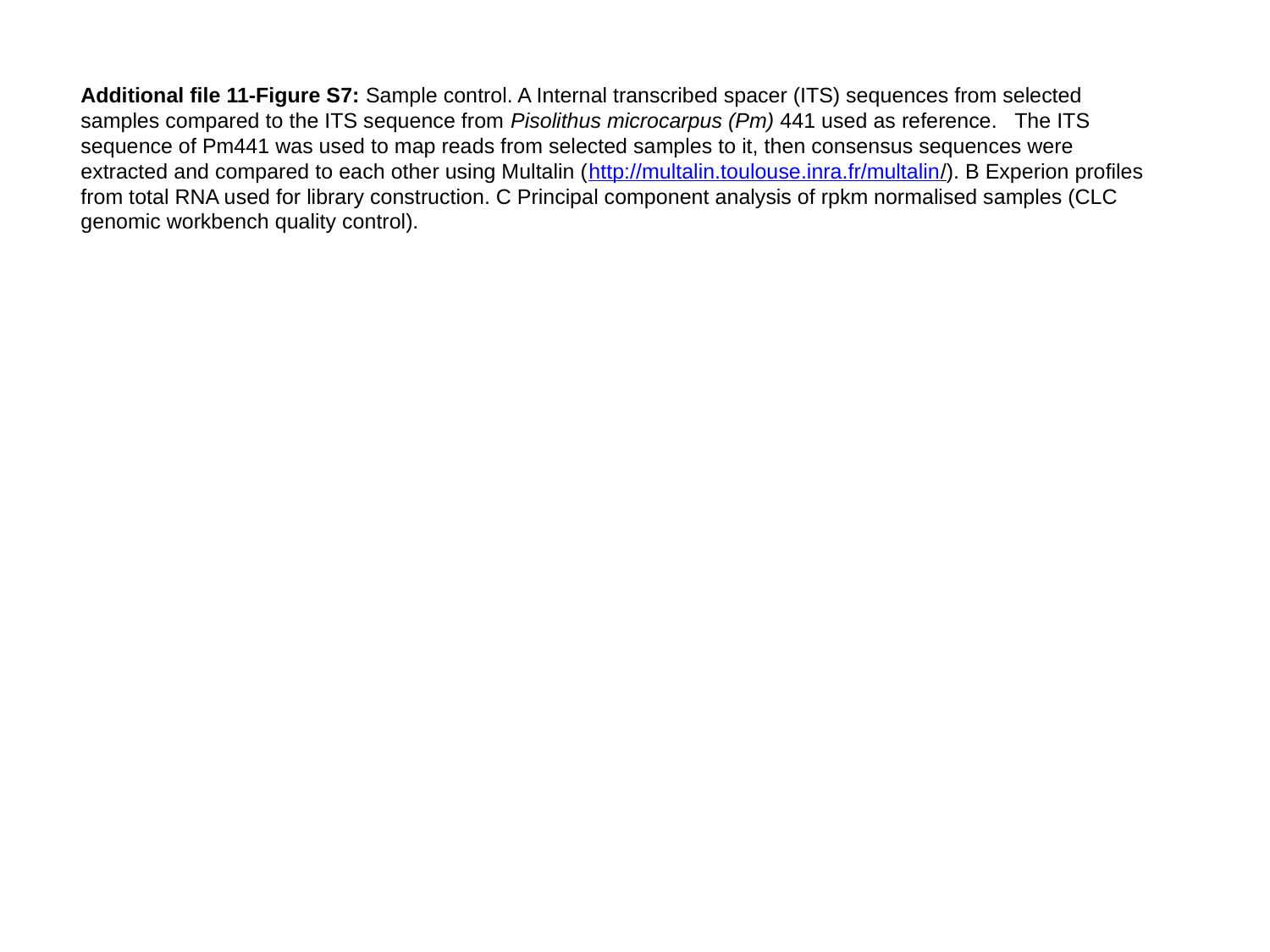

Additional file 11-Figure S7: Sample control. A Internal transcribed spacer (ITS) sequences from selected samples compared to the ITS sequence from Pisolithus microcarpus (Pm) 441 used as reference. The ITS sequence of Pm441 was used to map reads from selected samples to it, then consensus sequences were extracted and compared to each other using Multalin (http://multalin.toulouse.inra.fr/multalin/). B Experion profiles from total RNA used for library construction. C Principal component analysis of rpkm normalised samples (CLC genomic workbench quality control).
